# Accessing Mitochondrial Protein Import in Living Cells by Protein Microinjection

**DOI:** 10.3389/fcell.2021.698658

**Published:** 2021-07-07

**Authors:** Andrey Bogorodskiy, Ivan Okhrimenko, Ivan Maslov, Nina Maliar, Dmitrii Burkatovskii, Florian von Ameln, Alexey Schulga, Philipp Jakobs, Joachim Altschmied, Judith Haendeler, Alexandros Katranidis, Ivan Sorokin, Alexey Mishin, Valentin Gordeliy, Georg Büldt, Wolfgang Voos, Thomas Gensch, Valentin Borshchevskiy

**Affiliations:** ^1^Research Center for Molecular Mechanisms of Aging and Age-Related Diseases, Moscow Institute of Physics and Technology, Dolgoprudny, Russia; ^2^Environmentally-Induced Cardiovascular Degeneration, Central Institute of Clinical Chemistry and Laboratory Medicine, Medical Faculty, University Hospital and Heinrich-Heine-University Düsseldorf, Düsseldorf, Germany; ^3^IUF–Leibniz Research Institute for Environmental Medicine, Düsseldorf, Germany; ^4^Molecular Immunology Laboratory, Shemyakin & Ovchinnikov Institute of Bioorganic Chemistry, Russian Academy of Sciences, Moscow, Russia; ^5^Institute of Biological Information Processing (IBI-6: Cellular Structural Biology), Forschungszentrum Jülich, Jülich, Germany; ^6^Institute of Protein Research, Russian Academy of Sciences, Pushchino, Russia; ^7^Belozersky Institute of Physico-Chemical Biology, Lomonosov Moscow State University, Moscow, Russia; ^8^Institute of Biological Information Processing (IBI-7: Structural Biochemistry), Forschungszentrum Jülich, Jülich, Germany; ^9^JuStruct: Jülich Center for Structural Biology, Forschungszentrum Jülich, Jülich, Germany; ^10^Institut de Biologie Structurale (IBS), Université Grenoble Alpes, CEA, CNRS, Grenoble, France; ^11^Institute of Biochemistry and Molecular Biology (IBMB), Faculty of Medicine, University of Bonn, Bonn, Germany; ^12^Institute of Biological Information Processing (IBI-1: Molecular and Cellular Physiology), Forschungszentrum Jülich, Jülich, Germany

**Keywords:** mitochondria, mitochondrial protein import, microinjection, fluorescence microscopy, GFP, SNAP-tag

## Abstract

Mitochondrial protein biogenesis relies almost exclusively on the expression of nuclear-encoded polypeptides. The current model postulates that most of these proteins have to be delivered to their final mitochondrial destination after their synthesis in the cytoplasm. However, the knowledge of this process remains limited due to the absence of proper experimental real-time approaches to study mitochondria in their native cellular environment. We developed a gentle microinjection procedure for fluorescent reporter proteins allowing a direct non-invasive study of protein transport in living cells. As a proof of principle, we visualized potential-dependent protein import into mitochondria inside intact cells in real-time. We validated that our approach does not distort mitochondrial morphology and preserves the endogenous expression system as well as mitochondrial protein translocation machinery. We observed that a release of nascent polypeptides chains from actively translating cellular ribosomes by puromycin strongly increased the import rate of the microinjected pre-protein. This suggests that a substantial amount of mitochondrial translocase complexes was involved in co-translational protein import of endogenously expressed pre-proteins. Our protein microinjection method opens new possibilities to study the role of mitochondrial protein import in cell models of various pathological conditions as well as aging processes.

## Introduction

The vast majority of proteins in eukaryotic cells are produced in the cytoplasm. Two-thirds of cellular proteins by number (Juszkiewicz and Hegde, 2018) and 40% of cellular proteins by mass (Itzhak et al., 2016) are located in subcellular compartments (ER, mitochondria, nucleus, plasma membrane etc.). The protein sorting machinery serves a vital role in the cell life. Protein mislocalization results in protein aggregation in cytoplasm, which links it to Alzheimer’s and Parkinson’s diseases (Goedert, 2015). However, direct studies of protein transport inside living cells face a number of obstacles–mainly the requirement of being non-invasive and simultaneously using unmodified version of the protein to more closely match native conditions. Usually the protein of interest is introduced into cells in a form of DNA by transfection followed by translation by cell machinery. However, it is not possible to study protein transport kinetics with this approach. To study the rate of protein transport inside living cells, the following requirements should be fulfilled: (i) fast (compared to protein sorting machinery speed) delivery of protein into the cell, (ii) low fluorescence background signal, (iii) usability in adherent cells allowing microscopic observations. The delivery of a recombinant protein into cells can be achieved by various approaches, such as physical membrane disruption, protein modifications, or usage of nanocarriers (Du et al., 2018). Protein modifications (i.e., hypercharge, cell penetrating peptides, or poly-cysteine motifs) likely affect the protein behavior inside the cell, while nanocarriers require complicated engineering to avoid endosomal entrapment. Additionally, protein presence in the media around the cell, as is also the case for physical methods such as electroporation and optoporation, generates a fluorescence background signal compromising protein detection inside cells and lacks a clear temporal starting point. Microinjection of proteins gets around all these obstacles and hence is the method of choice for introduction of exogenous proteins in controllable fashion directly into the cytosol.

Protein microinjection has sporadically been used since the 1980s. Cytoskeleton rearrangements (Feramisco, 1979; Kreis and Birchmeier, 1982; Wiegers et al., 1991), nuclear import (Rosorius et al., 1999), protein binding (Phillip et al., 2012), the role of glycolysation (Rondanino et al., 2003), or blocking protein function by antibodies (Gorbsky et al., 1998; Keppeke et al., 2015) are among the very diverse biological problems studied with protein microinjection into living cells. With the increasing availability of super-resolution fluorescence microscopes, the direct introduction of highly fluorescent proteins (FPs) (preferably labeled with bright organic dyes) into cells became applicable for single particle tracking–to directly observe nuclear import (Dange et al., 2008) or to study formation of protein complexes by smFRET (Sakon and Weninger, 2010). In addition, the almost instantaneous nature of protein delivery into the cytoplasm by protein microinjection enables the observation of protein redistribution on a minute to hour time scale, which is especially interesting for protein import into cell organelles and other subcompartments.

Here we developed a procedure for gentle microinjection of plasmids and recombinant proteins into living cells. Mitochondrial protein import serves as an example, that our injection protocol is sufficiently non-invasive to preserve both the delicate mitochondria protein transport machinery as well as the cell viability in general. As a consequence, our method enabled us to obtain new mechanistic insight into the translocation process of mitochondrial proteins.

Almost all proteins localized in mitochondria are imported by a sophisticated import machinery. In mammalian cells, only 13 mitochondrial proteins are produced inside the mitochondria while the remaining vast majority of 1000–1500 proteins are expressed externally (Anderson et al., 1981; Pagliarini et al., 2008), translated at cytosolic ribosomes and later imported into the organelle. Protein import into the mitochondria requires the recognition of specific amino acid mitochondrial targeting sequences (MTS) of the precursor protein (pre-protein) by the respective translocase complexes.

Experiments performed *in vitro* on isolated mitochondria established that mitochondrial protein import can function in a purely post-translational manner and does not necessarily need the cytosolic co-factors (Becker et al., 1992). Under saturating pre-protein concentrations, rather high import rates can be achieved (Lim et al., 2001), showing that they are sufficient to avoid the accumulation of mitochondrial proteins in the cytoplasm. Experiments performed in living cells have pointed to a large variety of additional cytosolic factors affecting the targeting of mitochondrial proteins in the post-translational import (Becker et al., 2019), in particular molecular chaperones Hsp70 and Hsp90 (Young et al., 2003). A major argument for a post-translational mechanism of mitochondrial protein import has been the absence of a single specific ribosome-related regulatory and a targeting factor like the signal recognition particle in the case of protein import into ER (Walter et al., 1981).

However, co-translational mechanism is also convincingly supported by the existing data. Early microscopic data exhibited the presence of ribosomes attached to the outer mitochondrial membrane (OMM) (Kellems et al., 1974). Recently, polysomes directly attached to the translocase of the outer membrane (TOM) complex were observed *in vitro* by electron cryo-tomography (Gold et al., 2017). No experimental approach has been able to distinguish between those two mechanisms within living cells.

Here we show that our microinjection approach of exogenously produced proteins into living cells is a valuable tool in this respect. By injecting the fluorescent MTS-SNAP-tag protein, we show its mitochondrial import, which disappears with the deletion of MTS or the breakdown of the mitochondrial potential. When protein is injected into cells pre-treated with translation inhibitors–puromycin (PUR) or cycloheximide (CHX)–we detected their different effects on the mitochondrial import rate. Our results imply that around 50% of all TOM complexes are constantly occupied by co-translationally transported pre-proteins in living cells at rest. We believe that microinjection is a valuable tool not only for the studies of mitochondrial protein import in knock-out systems, disease, or aging models, but also as a method to investigate protein transport dynamics in other cellular organelles (see also Pelham et al., 1996; Rosorius et al., 1999).

## Materials and Methods

### Plasmid Preparation

pMC MTS-EmGFP and pMC MTS-Dendra2 plasmid were produced from pMC plasmid containing an MTS by restriction and insertion of the DNA-sequence encoding EmGFP and Dendra2, respectively. The gene of *Saccharomyces cerevisiae* SUMO was composed of *Escherichia coli* class II codons (Hénaut and Danchin, 1996) with the use of the DNA Builder software (The University of Texas Southwestern Medical Center at Dallas, USA). The nucleotide sequence at the 5′-end was additionally optimized using the RNA WebServer (Institute for Theoretical Chemistry, University of Vienna, Austria) in order to reduce the probability of RNA hairpin formation. The designed gene was synthesized by PCR from overlapping oligonucleotides (Stemmer et al., 1995) designed by means of DNA Works (Hoover and Lubkowski, 2002). The synthesis of oligonucleotides was purchased at Eurogen JCS (Moscow, Russia). The SUMO-tag was fused via overlap extension PCR with the MTS-EmGFP gene. The resulting SUMO-MTS-EmGFP PCR fragment was added via ligation into the Pet15b expression vector between the *Xba*I/*Xho*I sites. To obtain plasmid with the MTS-SNAP-tag, the EmGFP fragment was exchanged by the *Age*I/*Xho*I restriction followed by ligation of the SNAP-tag PCR fragment, resulting in SUMO-MTS-SNAP-tag chimera. The SUMO-SNAP-tag was also produced with overlap extension PCR and the following ligation into Pet15b plasmid between the *Xba*I/*Xho*I sites. All plasmids were verified by DNA sequencing for correct gene presence.

### Protein Expression

Transformed with the expression vector Pet15b containing either SUMO-MTS-EmGFP, SUMO-MTS-SNAP-tag or SUMO-SNAP-tag *E. coli* cells were grown in lysogeny broth (LB) medium up to OD_600_∼1.0 and induced with 1 mM IPTG (Helicon, Moscow, Russia). The cells were harvested after 3 h, centrifuged at 5000 × *g* for 10 min, and the pellet was frozen at −80°C. The cell lysis was performed either by a microfluidizer or ultrasound (depending on the amount of cells), in the lysis buffer (300 mM NaCl 50 mM NaH_2_PO_4_ pH = 7.0). The lysate was centrifuged at 10000 × *g* for 1 h, and the supernatant was applied onto affinity resin Ni-NTA (QIAGEN, Düsseldorf, Germany) in a column. After washing the column with the lysis buffer, the protein was eluted with 200 mM imidazole dissolved in the lysis buffer. Imidazole was removed by dialysis against the cell buffer: 130 mM KCl, 10 mM NaCl, 2 mM CaCl_2_, 20 mM NaHCO_3_ pH = 7.2. After dialysis, the solution was filtered through a 0.22 μm syringe filter (Merck Millipore, Darmstadt, Germany) and stored at 4°C.

### Label Preparation

BG-NH_2_ (New England Biolabs Inc., Ipswich, MA, USA) and NHS-Rho14 (ATTO-TEC GmbH, Siegen, Germany) were dissolved in DMF and mixed in 1:1 molar ratio in DMF, and 5× excess of triethylamine was added according to the manufacturer’s instructions. The reaction was performed overnight at 30°C. The purification was performed on SiO_2_ G-60 (Merck, Darmstadt, Germany) column with 50% DMF washing step and an elution with pure DMF, with functionalized SNAP dye being less soluble and slowly eluting in DMF. The dye was concentrated by evaporation in Centrivac (LabConco, Kansas City, MO, USA) using a vacuum pump ChemStar Dry (Gardner Denver, Milwaukee, WI, USA). The Surface-SNAP-ATTO594 and the SNAP-TMR-STAR dyes were obtained from New England Biolabs Inc. (USA).

### Protein Labeling and Purification

The dyes were added to purified 6His-SUMO-MTS-SNAP-tag protein in 1:1 molar ratio and left for 1 h at 30°C. After that, the protein solution was centrifuged to remove the precipitates and the non-bound dye was removed by a solution exchange through Amicon Ultra 30 kDa centrifugal filter (Merck Millipore, Darmstadt, Germany). The last step was repeated 3–5 times. After all the non-bound dye was removed, the His-tagged protease ULP-1 was added at a ratio of 1:500 to the SUMO-MTS-SNAP-tag protein (concentration determined by OD_280 nm_ measurement) for 1 h at 30°C. Afterward, the protein was again centrifuged to remove the precipitate and passed through the Ni-NTA column, with the MTS-SNAP-tag protein flowing through freely and 6His-SUMO staying bound on the column. The flow through was collected, concentrated in Amicon Ultra 10 kDa centrifugal filter (Merck Millipore, Darmstadt, Germany), the protein concentration and the labeling efficiency were determined by spectrophotometry using the ratio of OD_max label_/OD_280 nm_. The labeled protein was stored at 4°C for up to 2 weeks. The same procedure was followed for the SNAP-tag protein (without MTS) and the MTS-EmGFP. The labeling procedure was omitted for the last one. The protein quality was controlled by SDS PAGE (see Supplementary Figure 1B).

### Microinjection

Microneedles were prepared from capillaries with an outer/inner diameter of 1.2/0.94 mm (Harvard Apparatus, Cambridge, UK) in Sutter P-2000 (Sutter instruments, Novato, CA, USA). The protein was centrifuged at 20000 × *g* for 1 h before injection to remove small aggregates. The protein solution was back loaded into the needle, and the needle was installed into the micromanipulator InjectMan (Eppendorf, Hamburg, Germany). The micromanipulator was installed on the microscope allowing for immediate fluorescence microscopy after the injection and was connected to the microinjector FemtoJet (Eppendorf, Germany). The microinjection was performed at 20–30 hPa injection pressure, 0.1 s injection time, and a compensatory pressure of 20–25 hPa. Preliminary experiments were performed to estimate the injection volume. The fluorescence intensity of the diluted protein solution was measured using the same microscopy settings (laser power, gain, objective) as with the injection experiments. At a dilution of ca. 50 times the fluorescence intensity was comparable to the median intensity in the cells after injection of the labeled MTS-SNAP-tag, thus the injected volume was estimated to be 1–4% of the cell volume, fluctuating between the cells due to variation in the injected volume and the cell volume.

### Cell Culture

HeLa and HEK293 MTS-Dendra2 cells were grown in 25 cm^2^ flasks (Corning, Flintshire, UK) in DMEM (Gibco, Waltham, MA, USA) with 10% FBS (Gibco, USA) and PenStrep antibiotic (Gibco, USA). For the microscopy experiments, the cells were grown in a 35 mm μ-dish (Ibidi, Graefeling, Germany) to 40–60% confluency. If additional reagents were used, they were added 20 min beforehand to reach the following concentrations: PUR (Applichem, Darmstadt, Germany) 20 μg/ml, (Applichem, Germany) 100 μg/ml, Carbonyl Cyanide 3-ChloroPhenylhydrazone (CCCP) (Sigma Aldrich, Darmstadt, Germany)–50 μM. The Mitotracker Orange (MTOrange) (MitoTracker^TM^ Orange CM-H_2_TMRos, Thermo Fisher Scientific, Waltham, MA, USA) staining in the HeLa cells was performed with 100–200 nM concentration for 2–3 min. MTGreen (MitoTracker^TM^ Green FM, Thermo Fisher Scientific, USA) staining was performed with 500 nM concentration for 15 min. For the HEK293 MTS-Dendra2 cells no additional staining was used.

### HEK293-Dendra2 Line Generation

#### Cloning of a Lentiviral Expression Vector for Dendra2 Targeted to the Mitochondria

The plasmid pCMV/myc/mito was cut with *Sal*I and *Not*I to retain the MTS from subunit VIII of human cytochrome c oxidase and the C-terminal myc epitope tag as vector backbone. The coding sequence for Dendra2 was amplified from a plasmid clone and inserted into this backbone using the Gibson Assembly^®^ Cloning Kit (New England Biolabs, Frankfurt, Germany); the primer sequences are available upon request. The Dendra2 coding region in the resulting plasmid was verified by DNA sequencing to exclude point mutations due to nucleotide misincorporations during the amplification procedure. From this plasmid, part of the cytomegalovirus immediate early promoter and the complete MTS-Dendra2-myc coding region were excised with *Nde*I and *Xba*I and transferred to pLenti-FLAG-Trx-1 (Goy et al., 2014) cut with the same restriction enzymes to generate pLenti-MTS-Dendra2-myc.

#### Generation of a HEK293 Cell Clone Stably Expressing Dendra2 Targeted to the Mitochondria

5 × 10^4^ HEK293 cells were seeded on a 35 mm tissue culture dish and transduced with the MTS-Dendra2-myc lentiviral particles, as previously described (Goy et al., 2014), using a multiplicity of infection of approximately 10. Starting 6 days after the transduction, the cells were subjected to selection with 5 μg/ml PUR until all cells in a non-transduced control were dead. During selection procedure, cells were kept in a 1:1 mixture of the complete growth medium and the conditioned medium from an exponentially growing HEK293 cell culture. Single clones of the transduced cells were obtained by limited dilution (McFarland, 2000). Therefore, the transduced, selected cells were seeded in a 96-well tissue culture plate, with an average of 0.5 cells per well, in the same medium mixture as before. Successfully growing clones were analyzed by flow cytometry to assess monoclonal origin, and the exclusive mitochondrial localization of MTS-Dendra2 was verified by fluorescence microscopy. One of the clones was used for all further studies.

### Fluorescence Microscopy

The fluorescence microscopy was performed on an inverted laser-scanning confocal fluorescence microscope based on LSM780 (Zeiss, Jena, Germany). The 35 mm glass-bottom imaging dishes (Ibidi, Germany) were kept in an incubator maintaining 37°C, 5% CO_2_, 100% humidity (Tokai Hit, Shizuoka, Japan) that was mounted on the microscope stage. After the injection procedure, time-lapse imaging was performed in confocal fluorescent microscopy λ-mode using a 34-channel QUASAR detector (Zeiss, Germany) set to the appropriate spectral range depending on the dyes. For excitation, a 488 nm or 561 nm laser was used simultaneously with a 633 nm laser to excite the MTS-Dendra2 or MTOrange and the MTS-SNAP-Rho14/SNAP-Rho14 respectively. The injection experiments of MTS-EmGFP with the MTOrange labeling were done with 488 nm and 561 nm laser excitation. All the experiments were conducted with 1024 × 1024 (141 × 141 μm) image size using 100× (NA = 1.46, oil immersion) objective. The autofocus functionality of the LSM780 was utilized to the same z-plane using laser reflection on the sample dish glass surface before every image was taken. Afterward spectral unmixing was performed in the ZEN software (Zeiss, Germany) using saved spectra from the non-injected cells (MTOrange or MTS-Dendra2) and a droplet of a pure protein solution before the injection (MTS-EmGFP or MTS-SNAP-tag protein), respectively. dSTORM single molecule localization based super-resolution fluorescence microscopy was performed with the ELYRA.PS1-module of the Zeiss LSM780 microscope in TIRF-mode with iXon 997 (Andor, UK) camera with 256 × 256 px resolution (2 × 2 binning), with 20 ms frame for 30 s and excitation by 6 mW 642 nm laser.

### *In vitro* Mitochondrial Protein Import Assays

Import into isolated mitochondria was essentially performed, as described (Cenini et al., 2016). Briefly, the intact mitochondria were isolated from the cultured HeLa cells by differential centrifugation under isotonic buffer conditions. The radiolabeled pre-protein Su9-DHFR was generated by *in vitro* transcription and translation in reticulocyte lysate in the presence of ^35^[S]-methionine. The radiolabeled pre-proteins were added to the isolated energized mitochondria (25 μg total mitochondrial protein per lane) and incubated for up to 30 min at 30°C. To assess complete translocation, the import reactions were divided, and one half was treated with 50 μg/ml proteinase K (Merck, Darmstadt, Germany) to remove all non-imported pre-proteins. The mitochondria were then re-isolated, and their protein content was analyzed by SDS-PAGE and autoradiography. For import into semi-intact whole cells, the cultured HeLa cells were harvested (0.25 million cells per lane) and treated with 0.005% digitonin for 5 min at 25°C in the import buffer (250 mM sucrose, 20 mM HEPES pH 7.6, 80 mM KOAc, 5 mM Mg(OAc)_2_. The permeabilized cells were re-isolated by centrifugation for 10 min at 12000 × *g* at 4°C and gently resuspended in the import buffer containing 5 mM glutamate, 5 mM malate, 1 mM DTT, and 10 mM K_3_PO_4_. The import reaction was started immediately by the addition of the radiolabeled pre-protein, as described above. The pre-treatments with translational inhibitors puromycin (f. c. 20 μg/ml) and cycloheximide (f. c. 100 μg/ml) (Merck, Darmstadt, Germany) were performed after harvesting of the cells in the import buffer for 30 min at 37°C.

### Data Processing

The fluorescent images were manually segmented in Fiji (Schindelin et al., 2012) by a polygon tool into the regions with single, separated cells. In individual cells, a mitochondrial mask was created from the image of the used mitochondrial marker (MTS-Dendra2 or MTOrange). Next, we determined the average fluorescence intensity in the mitochondria and in the whole cell. The two values allow quantification of relative amounts of FP in mitochondria and whole cell at any given time point, since they are independent of the quite significant movements of cells and mitochondria within cells during the experiment (up to 90 min). To normalize for photobleaching and to remove the influence of the out-of-plane fluorescence signals from the cellular environment of the mitochondria, we calculated the ratio of the signal inside mitochondria to the signal of the cell. Time-series of this ratio reflects the time-dependent concentration of the imported protein inside mitochondria and are shown in Figure 5A. To determine the rate of import, time-series were linearly fitted in the time range 5–60 min for each cell, and the slopes were plotted in a box chart, as shown in Figure 5B. Super-resolution data analysis and image generation was done in thunderSTORM (Ovesný et al., 2014) plugin for Fiji.

### Statistics

Normal distribution for all data sets was confirmed by the Shapiro–Wilk test; homogeneity of variances (from means) between groups was verified by Levene’s test. Pairwise comparisons were performed with two-sided, unpaired Student’s *t*-tests on raw data. Multiple comparisons were performed using one-way ANOVA with the *post hoc* Tukey LSD test in Origin (OriginLab, Northampton, MA, USA).

### Polysome Profiling of PUR- and CHX-Treated Cells

HEK293 cells were pre-treated with PUR (20 μg/ml in DMEM) and CHX (0.1 mg/ml in DMEM) for 30 min at 37°C, similarly to the used treatment procedure in microinjection experiments.

HEK293T cell were washed twice with cold PBS containing 0.1 mg/ml CHX, then scrapped and resuspended in 1 ml PBS with CHX. All following procedures were carried on 4°C. Cells were collected in 2 ml microtubes and centrifuged for 5 min at 600 × *g*. The pellet was resuspended in 100 μl of lysis buffer (50 mM HEPES KOH pH 7.5; 2 mM MgCl_2_; 150 mM KCl, 1 mM DTT; 1× Complete Mini protease inhibitor cocktail, EDTA-free; 1% Triton X100; 0.1 mg/ml CHX) and incubated for 10 min. Debris from lysate were removed by centrifugation (10 min, 10000 × *g*), supernatant was transferred into a new microtube, then resuspended and frozen in liquid nitrogen.

Obtained lysates were loaded on 3.7 ml of 15–30% sucrose density gradient and sedimented at 45000 rpm for 40 min in a Beckmann SW55Ti rotor at 4°C. Sucrose gradients were formed using home-made system [described by Kopeina et al. (2008)], and contained 25 mM HEPES-KOH pH 7.6; 5 mM MgCl2; 100 mM KCl and 0.01 mg/ml CHX. Sedimentation profiles were recorded by Uvicord SII flow-photometer (LKB, Stockholm, Sweden) connected to E-24 (L-Card) AD-converter and PowerGraph (DISoft, Moscow, Russia) software. Blank profile was subtracted from the sample profiles.

## Results

### Microinjection of MTS-EmGFP Into Living Cells

**FIGURE 1:**
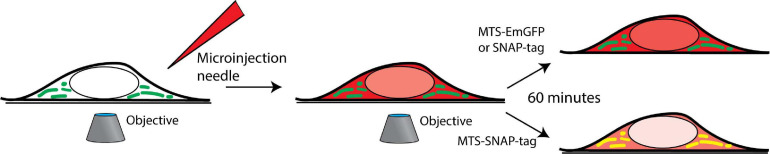
Microinjection is performed on the cells in the field of view of an inverted microscope, and time-lapse imaging is performed. The cells are grown in a 35 mm glass-bottom imaging dish and placed in an incubator mounted on an inverted confocal fluorescence microscope. The microinjection of the protein (red) is performed, and co-localization (yellow) with the mitochondria (green) is tracked over time.

**FIGURE 2:**
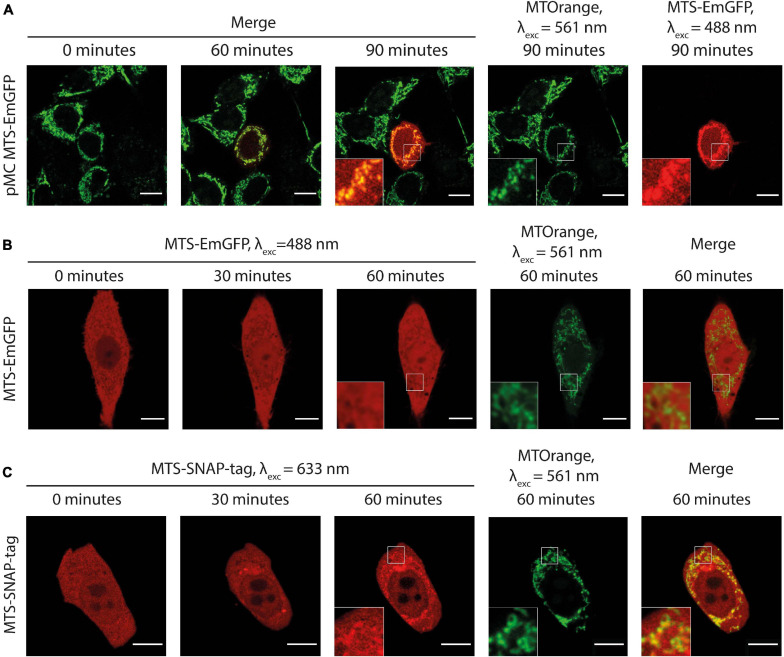
**(A)** Time-lapse microscopy of MTS-EmGFP (red) expression after the injection of the expression vector pMC-MTS-EmGFP in HeLa cell in the center of the image. MTS-EmGFP fluorescence is observed first at ca. 60 min and intensifies for several hours. The mitochondrial network labeled by MTOrange (green) appears normal at all times after the injection, and the newly synthesized and matured MTS-EmGFP localizes in the mitochondria. **(B)** Time-lapse microscopy of the injected MTS-EmGFP protein distribution in the HeLa cells. The MTS-EmGFP (red) evenly distributes inside the cytoplasm and nucleus, while the mitochondria labeled by MTOrange (green) are visible as regions with a lower fluorescence intensity (“shadows”), which correlates well with the strong fluorescence at the corresponding pixels in the MTOrange image. **(C)** Time-lapse microscopy of injected MTS-SNAP-tag protein in HeLa cells. The MTS-SNAP-tag (red) fluorescence distribution shows structures with a higher fluorescence intensity spatially correlating with the MTOrange labeled mitochondrial network (green) from 30 min after the injection, seen more clearly 60 min after the injection. Scale bars: 10 μm.

We developed an experimental procedure for microinjection of recombinant FPs into living mammalian cells with subsequent real-time microscopic detection of its cellular redistribution (see Figure 1). To establish the non-invasiveness of the injection for the mitochondria and the cell functioning, firstly, we injected the expression vector pMC MTS-EmGFP (emerald green FP), encoding EmGFP (Cubitt et al., 1999) targeted to the mitochondrial matrix, into adherent HeLa cells cultivated in a glass-bottom dish (Figure 2A and Supplementary Video 1). We used the MTS of cytochrome c oxidase subunit 8A (COX8A) to target FPs to the mitochondria. In such a way, we can monitor the cellular localization of FPs from the first minute up to several hours in the course of their intracellular expression. The mitochondria were labeled by MTOrange, which is spectrally well separated from MTS-EmGFP. After 1 h of incubation, a sufficient amount of MTS-EmGFP was produced, and its fluorescence signal built up in the mitochondria with a perfect match to MTOrange localization. MTS-EmGFP fluorescence appeared at later time points in the cytosol as well, however, in lesser amounts. This effect is caused by overexpression of MTS-EmGFP to very high levels, which the mitochondrial protein import machinery cannot handle properly. Additionally, we performed similar experiments with the pMC MTS-Dendra2 expression vector, Dendra2 (Gurskaya et al., 2006) being another member of the GFP-like FP family. In this case we observed several cells that divided hours after the plasmid injection that was followed by a boost of MTS-Dendra2 expression localized in the mitochondria (Supplementary Video 2). From these results we concluded that our injection procedure is sufficiently non-invasive: it does not disturb the mitochondrial health (as evidenced by the intact mitochondria shape and the functional protein import machinery) and the protein expression of the cell (as evidenced by the expression from the plasmid) in the course of the experiment. Even vital cell functions such as cell division were not compromised by microinjection.

With the established experimental parameters for microinjection, we moved on to the injection of recombinant FP. FPs were expressed in *E. coli* as part of a chimeric protein (Supplementary Figure 1A). The FP core is N-terminally fused with MTS followed by the SUMO protein. The role of SUMO is to protect MTS from proteolytic degradation during expression and purification (Malakhov et al., 2004). The degradation is likely to be caused by the N-terminal pathway in *E. coli* and the unspecific exoproteases activity inside bacterial cells (Gonzales and Robert-Baudouy, 1996). SUMO is cleaved by ULP1 SUMO protease after purification leaving intact MTS at the N-terminus of FP (Mossessova and Lima, 2000). As FPs, we used an EmGFP and the SNAP-tag protein, a self-labeling variant of the human enzyme *O*^6^-alkylguanine DNA alkyltransferase (Juillerat et al., 2003).

We injected the purified recombinant MTS-EmGFP into adherent HeLa cells. As shown in Figure 2B and Supplementary Video 3, the injected MTS-EmGFP dispenses fast in the cytosol while equilibration with the nucleus is completed only after 30–60 min. The MTS-EmGFP does not enter the mitochondria for up to 2 h of the observation time, as can be clearly seen by low-intensity areas (later referred to as “shadows”). These “shadows” overlap to a large extent with the MTOrange staining and, therefore, were identified as mitochondria. The low but non-zero EmGFP-fluorescence observed in these mitochondrial regions was possibly caused by out-of-plane fluorescence from proteins in the cytoplasm surrounding the mitochondria. Some “shadows” have no counterpart in the MTOrange staining, which most likely correspond to parts of the endoplasmatic reticulum. The volume of the injected MTS-EmGFP solution can be estimated as 1–4% of the cell volume (see the “Materials and Methods” section for details), which is in the range of naturally occurring cell volume regulation and does not harm the cell significantly. The mitochondrial network remained intact and morphologically unchanged in the course of the experiment.

The absence of the mitochondrial protein import unambiguously shows that no post-translational transport is possible for MTS-EmGFP. To further explore this phenomenon, we performed import experiments using structurally similar radiolabeled MTS-Dendra2, into the mitochondria isolated from yeast with a well-characterized experimental setup (Becker et al., 2009). The same lack of import of MTS-Dendra2 was observed (see Supplementary Figure 2) in the standard *in vitro* import assay. We therefore connect the absence of import with the structure of GFP-like FPs. The highly stable mature form of β-barrel proteins cannot fit into Tom40 pore in the folded state and cannot be unfolded by protein translocation forces alone.

### Microinjection of MTS-SNAP-Tag Protein Into Living Cells

As β-barrel FPs are not suitable for studying protein transport in our experimental setup, we focused on a different fluorescent reporter protein, namely the SNAP-tag protein. A SNAP-tag moiety in the expression vector COX8A-SNAP-tag was previously used for labeling the mitochondria after cell transfection (Stephan et al., 2019). SNAP-tagged fluorescent reporter was efficiently imported into isolated yeast mitochondria in an *in vitro* setup before (Martin et al., 2006). The major advantage of the SNAP-domain in fusion proteins is its ability to self-label with almost any (functionalized) fluorophore at a single defined cysteine residue in the active center of the SNAP enzyme.

Similar to MTS-EmGFP, the MTS-SNAP-tag protein was expressed in *E. coli* as N-terminal fusion with the MTS and SUMO-protein (Supplementary Figures 1A,B). The SUMO-MTS-SNAP-tag protein was labeled by SNAP-Rho14 (λ_exc_ = 633 nm; λ_em_ = 645 nm). The SUMO-protein was removed before microinjection and the labeled MTS-SNAP-tag protein was injected into HeLa cells (Figure 2C). Soon after the injection (<2 min), the MTS-SNAP-tag protein was distributed homogeneously in the cytoplasm and with a lower concentration in the nucleus. 20–30 min after the injection, the MTS-SNAP-tag protein was concentrated in cytoplasmic structures most of which are identified as the mitochondria by the MTOrange staining (Figure 2C and Supplementary Video 4). The visible accumulation continues to rise even 2 h after the injection. However, we used a cutoff point of 90 min for the following experiments. In this time range, photobleaching, dynamic range limitations, and cellular movement do not interfere significantly with the observations.

In contrast to MTS-EmGFP, the MTS-SNAP-tag protein is unambiguously imported into the mitochondria of living cells in a post-translational fashion. To the best of our knowledge, this is the first visualization of post-translational mitochondrial protein import observed in real-time in living cells.

Although the Rho14 chromophore is considerably smaller compared to the SNAP-tag protein, we cannot exclude its influence on the protein import. Accordingly, we performed the experiments with other, commercially available, SNAP dyes: SNAP-Surface 594 and SNAP-Cell TMR-Star. The MTS-SNAP-tag protein labeled with either dye exhibited similar import into the mitochondria. However, a noticeable variation in the import time was observed, likely reflecting different labeling efficiency, but an influence of the dye’s molecular structure cannot be ruled out (Supplementary Figures 3A,B). In all further experiments, we used Rho14 since it is spectrally well separated from green/yellow fluorescence of the mitochondrial markers used in this study: MTOrange and later MTS-Dendra2 (see below).

A further improvement of the experimental setup is related to the mitochondria marker. The labeling efficiency of the available MitoTracker dyes for living cells utilizes the inner mitochondrial membrane (IMM) potential and varies under experimental conditions. For this reason, we created a HEK293 cell line stably expressing MTS-Dendra2 with mitochondrial localization (see the “Materials and Methods” section for details of cell line generation). This approach provides a more convenient, stable, and cell-to-cell reproducible labeling of the mitochondria. In particular, this staining retains after the IMM potential depletion by CCCP, a widely used uncoupler of mitochondrial oxidative phosphorylation due to the inability of the MTS-Dendra2 to exit mitochondria in contrast to MTOrange (Supplementary Figure 4).

**FIGURE 3:**
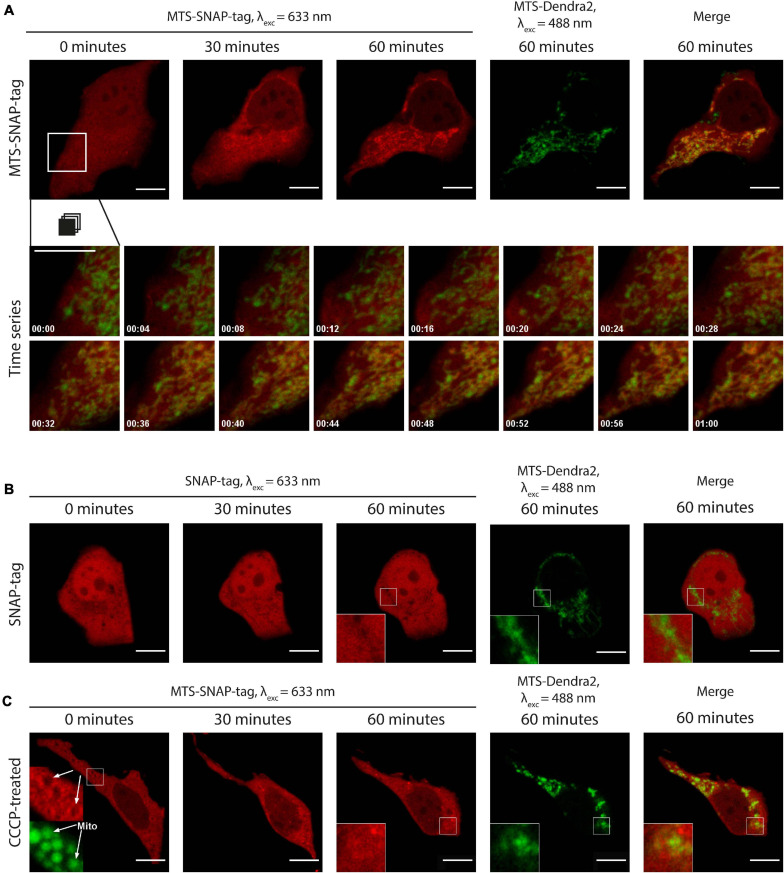
**(A)** Time-lapse microscopy of MTS-SNAP-tag protein redistribution in HEK293-MTS-Dendra2 cells after the injection. The MTS-SNAP-tag protein fluorescence (red) co-localizes with the mitochondria after 30 min. The mitochondria are labeled by the MTS-Dendra2 protein (green). The bottom row shows a time series of the selected region. The tracking time is given in minutes. **(B)** Time-lapse microscopy of the SNAP-tag protein redistribution (red) in the HEK293-MTS-Dendra2 cells after the injection, the “shadows” correspond to the mitochondria, as seen in the enlarged inset by comparison with the MTS-Dendra2 labeled mitochondria (green). **(C)** Time-lapse microscopy of the MTS-SNAP-tag protein redistribution after the injection in the HEK293-MTS-Dendra2 pre-incubated with 50 μM CCCP. The MTS-SNAP-tag protein (red) initially forms a brighter rim around the spherical mitochondria (green), indicated by white arrows in the enlarged insets in the first image (0 min). Scale bars: 10 μm.

Microinjection of the fluorescent MTS-SNAP-tag protein into HEK293-MTS-Dendra2 cells completely reproduced all import features observed in HeLa cells, demonstrating the suitability of a different cell type for mitochondrial protein import experiments in living cells (Figure 3A and Supplementary Video 5).

The experimental setup utilizing HEK293 with the MTS-Dendra2-labeled mitochondria and the injection of SNAP-tag labeled with Rho14 was used in all later experiments. To study the speed of mitochondrial protein import in living cells in each experiment, we collected confocal fluorescence images every 2 min after the injection typically for about 90–120 min after the injection. A time series of fluorescent images composes a “movie” of the MTS-SNAP-tag protein transport into the mitochondria (see Figure 3A and Supplementary Video 5). With this setup, one can clearly detect the import of MTS-SNAP-tag protein into mitochondria in the merged image by the disappearance of green and the concomitant appearance of yellow mitochondria at later time points, most pronounced between 20 and 50 min after the protein injection. The experiments described below were repeated, and all the described observations were reproduced for at least five cells.

To validate our experimental setup and to exclude potential artifacts of the protein purification and labeling, we produced the SNAP-tag protein without an MTS. The SNAP-tag protein was labeled with SNAP-Rho14 and injected into the cell (Figure 3B and Supplementary Video 6). Similarly, to the MTS protein variant injection, the SNAP-tag protein was evenly distributed in the cytoplasm right after the injection. However, no change in its spatial distribution was observed during the next 90 min. Instead, similar to MTS-EmGFP, the mitochondria were seen as “shadows” in the uniform SNAP-tag fluorescence in the cytoplasm. The “shadows” coincide well with the MTS-Dendra2 fluorescence of the mitochondria (see insets in Figure 3B). These “shadows” are a direct consequence of the exclusion of the SNAP-tag protein from the mitochondria.

To further elucidate the correct localization of the protein inside the mitochondria in a typical experiment we have performed dSTORM single molecule localization based super-resolution fluorescence microscopy of the living cells roughly 3 h after injection. We observed MTS-SNAP-tag protein in the mitochondrial matrix as can be deduced from the comparison of the same mitochondrial structures in fluorescence wide-field and super-resolved image (Supplementary Figure 5). Mitochondria cannot be resolved properly with conventional fluorescence microscopy methods, however, with dSTORM and other super-resolution fluorescence microscopies several details known from electron microscopy have been successfully visualized (see e.g., Appelhans et al., 2012; Klotzsch et al., 2015; Dlasková et al., 2018). The distribution of MTS-SNAP-tag protein fluorescence in the dSTORM images is central but much narrower when compared to the conventional wide-field fluorescence image. The resolution in our dSTORM images is greatly enhanced so that we can estimate the proper width of mitochondrial matrix in the range of 100–200 nm (Appelhans et al., 2012; Klotzsch et al., 2015) and confirm localization of MTS-SNAP-tag protein in mitochondrial matrix.

### Effects of Uncoupling of IMM Potential on MTS-SNAP-Tag Protein Import in Living Cells

The IMM potential is a major driving force for the import of mitochondrial pre-proteins. To assess the effects of uncoupling chemicals, dissipating the IMM potential, we injected the MTS-SNAP-tag protein into cells pre-treated with CCCP. Disrupting the mitochondrial potential, CCCP interferes with its tubular morphology (Legros et al., 2002). The mitochondria become spherical or elliptical, which we also observed in our experiment (Figure 3C and Supplementary Video 7). Right after the injection, the mitochondria appeared as “shadows” in the MTS-SNAP-tag image with a bright rim around them (see inset in Figure 3C at 0 min). It probably represents the MTS-SNAP-tag protein molecules that were attached to the mitochondrial surface via the TOM machinery but were not translocated through the IMM due to the absence of the potential. This feature disappears over time (in approx. 10 min). Seemingly, the cause is the recovery of the more elongated mitochondria shape after injection (Figure 3C at 60 min), which weakens the contrast due to the optical resolution limitations. The MTS-SNAP-tag was not efficiently imported into the mitochondria as compared to untreated cells. However, the MTS-SNAP-tag protein “shadows” were also visible to a lesser extent in the CCCP-treated cells than for SNAP-tag protein w/o MTS injected in non-treated cells. Therefore, we cannot exclude the possibility that a low amount of protein import still can occur in the absence of the membrane potential. An alternative explanation is that due to the change in the mitochondrial morphology (spheres/ellipses instead of the mitochondrial network), the mitochondrial “shadows” are less well resolved and more smeared in the case of the CCCP-treated cells compared to non-treated ones. In summary, protein accumulation inside the mitochondria under these conditions is almost negligible compared to protein import levels in untreated cells. Consequently, the mitochondrial potential is necessary to efficiently import pre-proteins in living cells.

### Influence of Ribosome-Nascent Chain Complex (RNC) State on Mitochondrial Protein Import in Living Cells

In our experimental setup, we use living cells that actively translate endogenous proteins, some of which are targeted to mitochondria. The injected reporter protein competes for mitochondrial import with the endogenous ones. We use PUR and CHX to prevent new protein production and consequently preclude import into the mitochondria. Both compounds are widely used translation inhibitors, but have different action mechanisms. PUR causes premature translation termination and nascent chain release from the ribosome (Nathans, 1964), whereas CHX stalls translation but preserves intact RNC (Ennis and Lubin, 1964).

**FIGURE 4:**
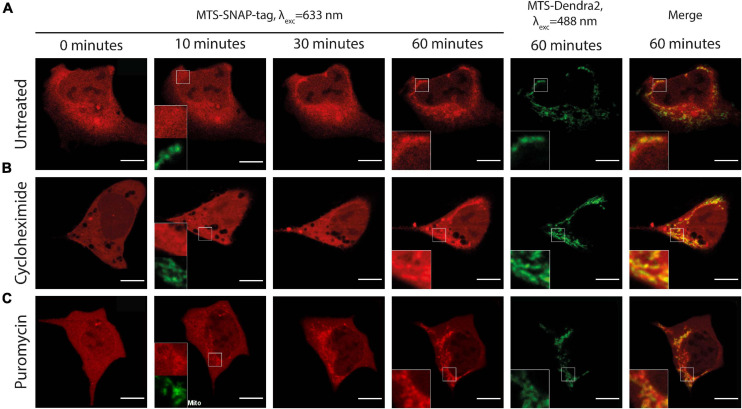
Time-lapse microscopy of the microinjected MTS-SNAP-tag protein (red) import into the mitochondria in HEK293 MTS-Dendra2 (green) cells under different conditions. **(A)** Untreated HEK293-MTS-Dendra2 cells. After 10 min, no noticeable MTS-SNAP-tag protein accumulation in the mitochondria can be seen (shown enlarged in the inset). MTS-SNAP-tag protein accumulation is seen after 60 min. (enlarged region in the insets). **(B)** HEK293-MTS-Dendra2 cell treated with 100 μg/ml CHX. After 10 min, no noticeable MTS-SNAP-tag protein accumulation in the mitochondria can be seen (shown enlarged in the inset). The MTS-SNAP-tag protein accumulation is seen similarly to the untreated cells (enlarged region in the insets) after 60 min. **(C)** HEK293-MTS-Dendra2 cell treated with 20 μg/ml PUR. After 10 min, MTS-SNAP-tag protein accumulation in the mitochondria can be seen (shown enlarged in the inset). After 60 min, the MTS-SNAP-tag protein accumulation results in brighter mitochondria as compared to the CHX-treated and untreated cells. Scale bars 10 μm.

We injected the Rho14-labeled MTS-SNAP-tag protein into HEK293-MTS-Dendra2 cells pre-treated for 30 min with either PUR or CHX. As shown in Figure 4, in both cases, we clearly observed protein import much alike as in the untreated cells. However, the rates of import were significantly different between the two. The mitochondrial protein import was faster in the PUR-treated cells–the Rho14 fluorescent signal localized to the mitochondria was already clearly visible as early as 10 min after the injection (Figure 4C and Supplementary Video 9). CHX had almost no effect on the mitochondrial protein import rate compared to untreated cells–the MTS-SNAP-tag protein import into mitochondria occurred on the time scale of 30–60 min (compare Figure 4B with Figure 4A, and Supplementary Video 8 with Supplementary Video 5).

We verified effectiveness of used concentrations and exposure times for PUR and CHX by performing polysome profiling using the same treatment conditions for HEK293 cells (Supplementary Figure 6).

**FIGURE 5:**
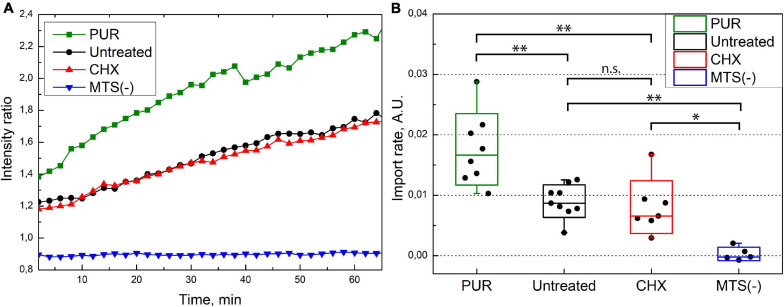
**(A)** MTS-SNAP-tag protein import into the mitochondria. Each line represents the ratio of average mitochondria fluorescence to cellular fluorescence over time averaged for 6–9 cells under similar conditions. The PUR treated cells (green) show a higher import speed compared to the CHX- and untreated cells (red and black, respectively). The SNAP-tag protein (blue) shows no import into the mitochondria. **(B)** Import rate under different conditions. The ratio of average fluorescence from mitochondria and whole cell of the MTS-SNAP-tag protein over time is linearly fitted between 5 and 60 min, and the slope coefficients are plotted. The boxes show a standard deviation, the whiskers indicate the range of values. 7/7/9 cells from three separate experiments were used for puromycin/cycloheximide/no treatment, respectively. For the SNAP-tag protein experiment, five cells were used from a single experiment. The significance level is given according to the one-way ANOVA with the *post hoc* Tukey LSD test: ^∗∗^*p* < 0.005, ^∗^*p* < 0.05, n.s.–not significant.

We quantified the rate of the mitochondrial protein import for untreated, PUR- and CHX-treated cells. We related the fluorescence intensity in mitochondria to that in the whole cell and used the ratio as a measure (see the “Materials and Methods” section for details) for the amount of imported protein. The increase in ratio with time is, therefore, proportional to the increase in the amount of imported protein. Figure 5A depicts the time series of the mitochondrial-to-cellular ratio of FP concentration averaged over all measured cells for a given condition. We treated our previously described results on the injection of SNAP-tag w/o MTS in the same way and used as a negative control in Figure 5A. We fitted the determined fluorescent-protein concentration ratio in the time interval of 5–60 min by linear equation and used the slope coefficient as quantitative measure of the import rate. The import rate for each cell is plotted in a box chart in Figure 5B. Consistent with the previous results, the import rate for SNAP-tag protein w/o MTS is close to zero. No differences in the mitochondrial protein import rates was detected between the untreated and CHX-treated cells (*p*≈0.96); however, the import rate in PUR-treated cells is ∼2-times faster compared to CHX-treated or untreated cells (*p*≈0.0008 and 0.0012, respectively). As described in the discussion, the different import rate for the PUR- and CHX-treated cells is likely connected to the RNC attached to the TOM complexes on the cytoplasmic side of the OMM.

### Effect of CHX and PUR on the *in vitro* Mitochondrial Protein Import

**FIGURE 6:**
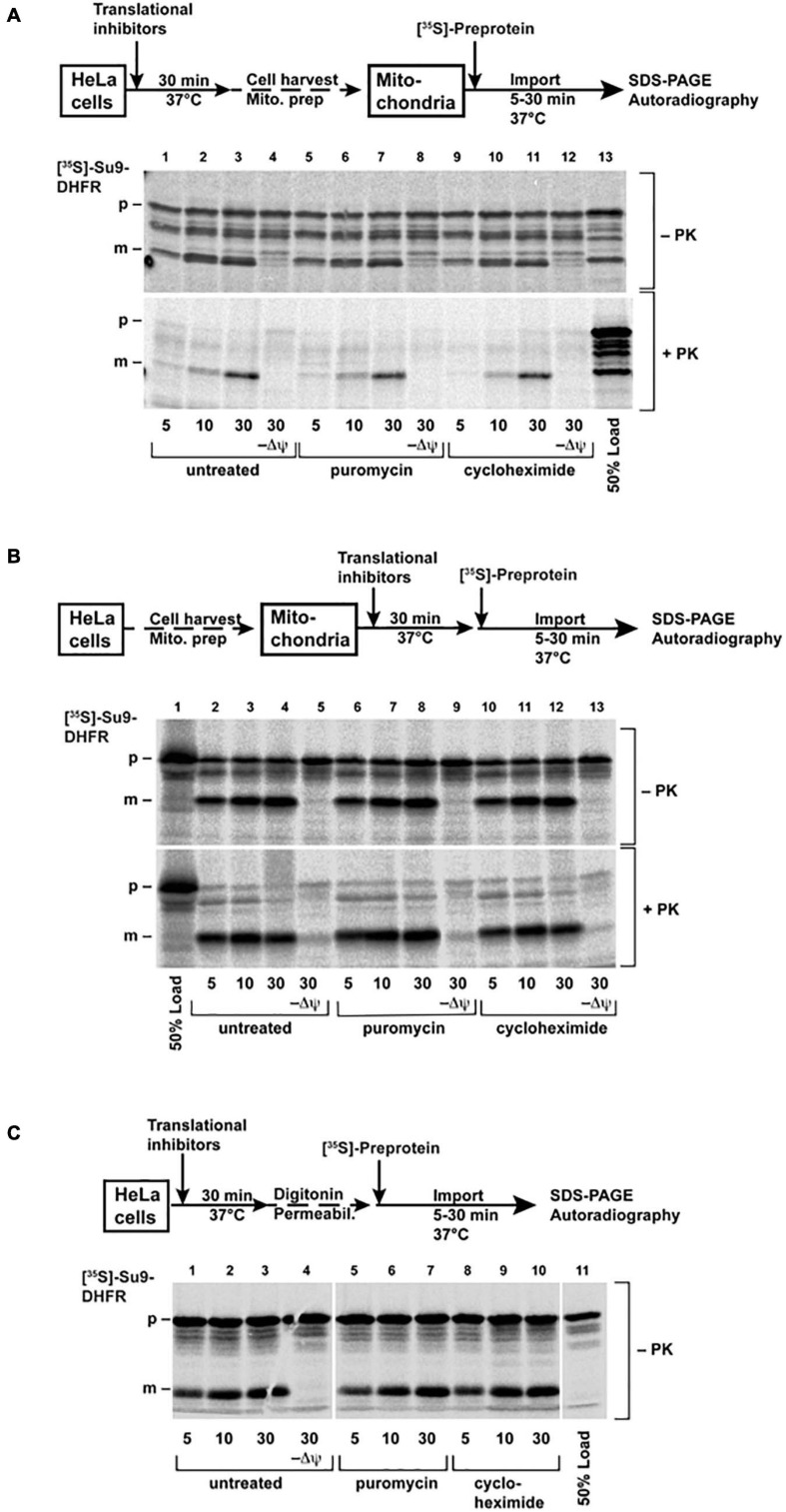
Import of the [^35^S]-labeled protein Su9-DHFR was performed as described in the “Materials and Methods” section. The cells **(A)** or the isolated mitochondria **(B)** were pre-treated before import with PUR (20 μg/ml) or CHX (100 μg/ml) for 30 min at 37°C. The import reactions were incubated for the indicated times. **(C)** The cells were permeabilized with digitonin after pre-treatment, and then import was performed directly without isolation of the mitochondria. In the control reactions (–Δψ), the inner membrane potential was depleted, as described. After import, the cells were treated with proteinase K, as indicated. The imported proteins were analyzed by SDS-PAGE and autoradiography (p: precursor form, m: mature form of Su9-DHFR). The schematic outlines of the experimental procedures are given.

To put the observations of the microinjection experiments into the context of the previous experimental work on the mitochondrial protein import, we also performed *in vitro* mitochondrial protein import experiments, which are typically based on a post-translational approach. Here, mitochondrial pre-proteins are synthesized in a cell-free system, in a radiolabeled form, and incubated with the isolated, intact, and energized mitochondria in an appropriate buffer system. As an import reporter protein, we used the well-established construct Su9-DHFR, consisting of a MTS derived from the subunit 9 of the mitochondrial Fo-ATP synthase from the mold *Neurospora crassa* and the cytosolic dihydrofolate reductase (DHFR) enzyme from the mouse as a cargo moiety. This pre-protein was synthesized and labeled with [S]^35^-Methionine by *in vitro* transcription and translation in rabbit reticulocyte lysate. The radiolabeled Su9-DHFR was efficiently imported *in vitro* into the mitochondria isolated from human HeLa cells, as expected (Figure 6A). The analysis of the radioactive proteins associated with the mitochondria by SDS-PAGE and autoradiography indicated an increasing amount of the processed form (p) over time. The p-form is generated by the matrix processing peptidase, indicative of the removal of the targeting sequence after translocation into the mitochondrial matrix. In addition, the imported radiolabeled pre-proteins were protected against degradation by the externally added protease K (PK) due to the still intact mitochondrial membranes. Typically for a successful import reaction, both processing and acquisition of protease resistance are dependent on the presence of an intact IMM potential, as shown in the control experiments where the mitochondria were pre-treated before import with inhibitors of oxidative phosphorylation and uncoupling chemicals (−Δψ). The post-translational import of Su9-DHFR under *in vitro* conditions appeared to be fast with detectable processing and translocation already in the time frame of minutes. A pre-treatment of the isolated mitochondria by CHX or PUR, in order to affect potentially bound ribosomes that had not been removed by the isolation procedure, did not change the import efficiency or kinetics.

In order to most genuinely reproduce the conditions of the living cells, we also pre-treated the intact HeLa cells in culture medium with the same amounts and times of CHX and PUR as were used in the injection experiments, isolated mitochondria from these cells, and repeated the import experiments using radiolabeled Su9-DHFR, as described above (Figure 6B). Again, no difference between the import rates in the mitochondria isolated from control and the pre-treated cells was observed.

In another experiment we also adopted a technical procedure similar to previously described for yeast cells (Laborenz et al., 2019), by using semi-permeabilized cells for mitochondrial protein import experiments. In this case, the elaborate mitochondria isolation procedure is circumvented, and any experimental manipulation of the cell system is reduced to a minimum. The permeabilization of the cell membrane was achieved by treatment of the cells harvested from the culture with the mild detergent digitonin under isotonic buffer conditions. The radiolabeled Su9-DHFR was added to this suspension of the permeabilized cells. After different incubation times, the cells were re-isolated, and the radioactive proteins were analyzed by SDS-PAGE and autoradiography (Figure 6C). Similar to the traditional import experiments with purified mitochondria, we observed fast processing and also protease resistance of the Su9-DHFR pre-protein, indicative of a successful and efficient mitochondrial protein import process. However, even under these conditions mimicking living cells, the presence of ribosomal inhibitors did not make a difference to the import reaction.

Taken together, these experiments indicate that in the *in vitro* setup of a mitochondrial protein import reaction, the post-translational import exhibits kinetics different from injection in living cells, since both translational inhibitors are unable to change the import reaction. In contrast, our novel approach demonstrates differences and thus, much closer resembles the genuine situation of mitochondrial protein import in living cells, particularly concerning translocation kinetics and the relationship between post- and co-translational import processes.

## Discussion

Current knowledge of protein import into mitochondria originates mainly from *in vitro* experiments on isolated intact mitochondria supplied with exogenously expressed precursor proteins. Direct visual observation of mitochondrial protein import in living cells was impossible for a long time due to the lack of adequate approaches. Isolated mitochondria lose their natural environment (cytosolic cofactors, involvement of the RNCs, microtubules, and other organelles) and also their native morphology, being transformed from a tubular network to separate spherical, double-bilayer vesicles. Although most principal processes–like oxidative phosphorylation–are preserved in the isolated mitochondria, some of their functional properties, including protein import, are likely to be compromised. Even in this state, co-translational import mechanism was also shown for the import of the enzyme fumarase into the isolated mitochondria (Knox et al., 1998). Fumarase was not imported into the organelle when expressed separately. In contrast, a significant amount of protein was detected inside the mitochondria when the fumarase pre-protein was translated in the presence of mitochondria when the polypeptide is able to directly engage the mitochondrial translocation machineries before the translation is completed or the protein is fully folded.

In living cells, indications for co-translational import has been observed initially due to mRNA localization (Egea et al., 1997) and ribosome clustering (Kellems et al., 1974) near OMM. Both localized mRNA sequencing (Fazal et al., 2019) and localized ribosome profiling (Williams et al., 2014; Vardi-Oknin and Arava, 2019) show a large amount of tightly associated and actively translated mRNA near the OMM. Some mRNAs are attached via nascent-chain of actively translating ribosomes, and dissociate from the OMM in the absence of either ribosome translation or mitochondrial potential. Therefore, active ribosomes are associated with the translocation machinery by the interaction of the nascent polypeptide chain with the TOM complex. Additional indirect evidence for co-translational import comes from genetic studies. The deletion of OM14 (a tail-anchored OMM protein) leads to a reduced association of ribosomes with OMM (Lesnik et al., 2014) while Tom20 deletion causes partial mRNA dissociation from OMM (Eliyahu et al., 2010). Taken all these findings together, both co- and post-translational processes have certain roles in mitochondrial protein transport. However, their relative importance in living cells under different conditions needs to be determined.

Here, we developed a setup for direct investigation of protein transport based on confocal fluorescence microscopy and microinjection of pre-expressed FPs into living cells. In our study, we preserved the natural state of cells, including functional expression machinery and undisturbed mitochondria in their natural state. To enable fluorescent detection of the import, we used different FPs N-terminally tagged with an MTS as a model for putative protein targeted to the mitochondria. The use of an MTS for targeted delivery of the desired proteins to the mitochondria is widely applied to deliver FPs for mitochondria labeling or to influence mitochondrial function (Hoffmann et al., 1994; Tkatch et al., 2017; Stephan et al., 2019).

In our first approach, we used EmGFP as a fluorescent reporter. However, the purified MTS-EmGFP showed no mitochondrial protein import after microinjection. A plausible reason is that the folded β-barrel cannot fit through Tom40 pore in the OMM–the β-barrel diameter is ∼30 Å, whereas the internal diameter of the Tom40 pore is ∼15 Å. Unfolding of EmGFP during transport through the Tom40 pore, which would enable transport, is also unlikely due to the known high stability of the β-barrel fold of FPs (Stepanenko et al., 2013). It should also be noted that although many successful experiments were performed with different proteins imported into isolated mitochondria, no import into the IMM or the mitochondrial matrix was ever shown for proteins with a stable β-barrel structure. Therefore, we switched to the SNAP-tag protein–an engineered variant of the enzyme O6-alkylguanine-DNA alkyltransferase, which is able to spontaneously form a covalent bond to virtually any fluorescent dye chemically fused with a benzylguanine. The purified and labeled MTS-SNAP-tag reporter showed successful import into mitochondria in living cells. The SNAP-tag size is close to that of EmGFP (23 and 27 kDa, respectively) and also will not fit through the Tom40 pore in its folded state (dimensions of ∼25 by 35 Å). However, the SNAP-tag has α + β-fold and is apparently more amenable to partial unfolding and transport through the Tom40 pore.

It is worth pointing out the seemingly slow import rate. Using the rate of cell multiplication in HEK293 cells one can estimate that in 25 h mitochondria mass should double. Mitochondrial proteins comprise roughly 6% of the total protein mass in one cell (assuming content is similar in HeLa and HEK293 cell lines) (Itzhak et al., 2016). As protein concentration in HEK293 cells is ∼150 mg/ml (Gillen and Forbush, 1999), total mitochondrial protein concentration is ∼10 mg/ml in the whole cell. Therefore, injected protein concentration (10 mg/ml diluted 50 times) can be estimated to be around 1/50th of the mitochondrial protein concentration inside the cell. Thus, assuming all else being equal, such protein amount should be imported in roughly 30 min. Our observations demonstrate slower protein uptake than expected. There can be several explanations for such a behavior: slowing down of import by existing protein translation (which we demonstrated in case of PUR treatment), protein being bound to elements of the cellular quality control machinery resulting in cleavage of MTS or other protein modifications that decrease the ability of injected proteins to be imported into mitochondria. Additionally, we have to note that an introduction of the excess mitochondrial pre-proteins in the cytoplasm can elicit certain cellular stress responses (Boos et al., 2019). The major responses to this pre-protein occur on the level of transcription, e.g., overexpression of chaperone or proteasome components. Both processes, however, need at least 30 min to contribute significantly to the experiment and therefore are too slow to affect significantly the observed time course of mitochondrial protein import in our experiments.

The mitochondrial uncoupling agent CCCP is known to stop the import of proteins into isolated mitochondria (Schleyer et al., 1982) and is widely used as an important negative control in mitochondrial protein import experiments. Consistent with that, CCCP-treated cells showed no noticeable protein import into the mitochondria in our experiments. It should be noted that in the presence of CCCP, the MTS-containing proteins can still interact with the import machinery of the OMM but will not be inserted by the translocase of the inner mitochondrial membrane (TIM). Hence, the MTS-SNAP-tag protein is initially recognized by the TOM machinery, which, however, lacks the driving force of the mitochondrial potential. Consequently, the polypeptides cannot be transported through the membrane, resulting in a higher concentration on the surface without any localization inside, seen as an initial brighter rim.

Puromycin and CHX are two commonly used translation inhibitors. Translation inhibition influences production of all cellular proteins, including the TOM complexes themselves. However, as the pre-treatment exposure for 30 min was very short compared to endogenous protein expression rates, the influence on the amounts of the TOM components or other mitochondrial proteins is negligible. Pre-treatment of the cells with these inhibitors before the injection of the protein caused remarkably different effects on the mitochondrial protein import reaction–CHX did not change the import rate significantly, whereas PUR increased it approximately two times. It sheds light on the long-standing question of whether post- or co-translational import prevails for mitochondria-targeted proteins in living cells. The translational inhibitors affect the internal expression of cellular proteins, including those of endogenous mitochondrial proteins. CHX “freezes” RNC attached to the OMM (Kellems et al., 1974; Gold et al., 2017) and, thus, clogs the TOM complexes CHX treatment should presumably result in a deceleration of mitochondrial protein import, if the recently reported increase of OMM-bound mRNA in the presence of CHX (Fazal et al., 2019), is provoked mainly by a larger amount of translating RNCs at the OMM. Localized translation in the direct vicinity of mitochondria has also previously been observed for HEK293 cells (Vardi-Oknin and Arava, 2019). We do not observe a loss of import speed between CHX-treated and untreated cells (Figure 5B). It might be explained by concurrent suppression of post-translationally imported protein production. Alternatively, the observed increase in the number of RNCs is not directly connected to TOM occupancy, but rather interactions of RNCs with other OMM proteins. On the other hand, PUR dissociates RNCs and also releases them from the mitochondrial surface (Fazal et al., 2019), if they interact via a newly translated protein. Hence, we conclude that the observed two-fold increased import rate of the injected proteins results from the removal of all RNCs attached to TOM complexes by PUR, which, upon removal, became available for post-translational mitochondrial protein import. Hence, we conclude that about 50% of all TOM complexes were occupied by co-translational import of the endogenous proteins. In living cells, post-translational and co-translational import of endogenous proteins compete with each other, and the rate of post-translational import is strongly affected by the occupation of the TOM complexes with RNC.

Our work provides an insight into mitochondrial protein import inside living cells, directly demonstrating not only import of exogenous proteins into mitochondria, but indirectly demonstrating the significant role of co-translational mitochondrial protein import. Our approach to study mitochondrial protein import provides a valuable tool to study protein import directly in living cells systems under different conditions, such as import machinery knock-out studies or in model systems of cardiovascular and neurodegenerative diseases prominently involving mitochondrial defects (Pickrell and Youle, 2015; Cenini et al., 2016).

## Data Availability Statement

The original contributions presented in the study are included in the article/Supplementary Material, further inquiries can be directed to the corresponding authors.

## Author Contributions

GB, TG, VB, WV, JH, and JA designed the experiments. AB produced the proteins, performed the microinjection experiments, fluorescence microscopy, and data processing. IO, NM, AK, and AS produced plasmids and helped with protein expression. FA, IM, PJ, JA, and JH generated HEK293 MTS-Dendra2 cell line. IM and DB helped with cell line maintenance, fluorescence microscopy, and data processing. IS performed the polysome profiling experiments. GB, VG, VB, TG, JH, JA, and AM acquired funding. TG and VB supervised the project. AB, VB, and TG analyzed the data and wrote manuscript with the impact from WV, JH, JA, GB, VG, AM, IO, AK, IM, and NM. All authors contributed to the article and approved the submitted version.

## Conflict of Interest

The authors declare that the research was conducted in the absence of any commercial or financial relationships that could be construed as a potential conflict of interest.
