# Supplementary material for: Accessing Mitochondrial Protein Import in Living Cells by Protein Microinjection
Source: Front Cell Dev Biol. 2021 Jul 7;9:698658. doi: 10.3389/fcell.2021.698658 (PMC8292824; doi:10.3389/fcell.2021.698658)
Supplement: Supplementary file 10 [file Data_Sheet_1.pdf]

Supplementary Material

1 Supplementary Figures

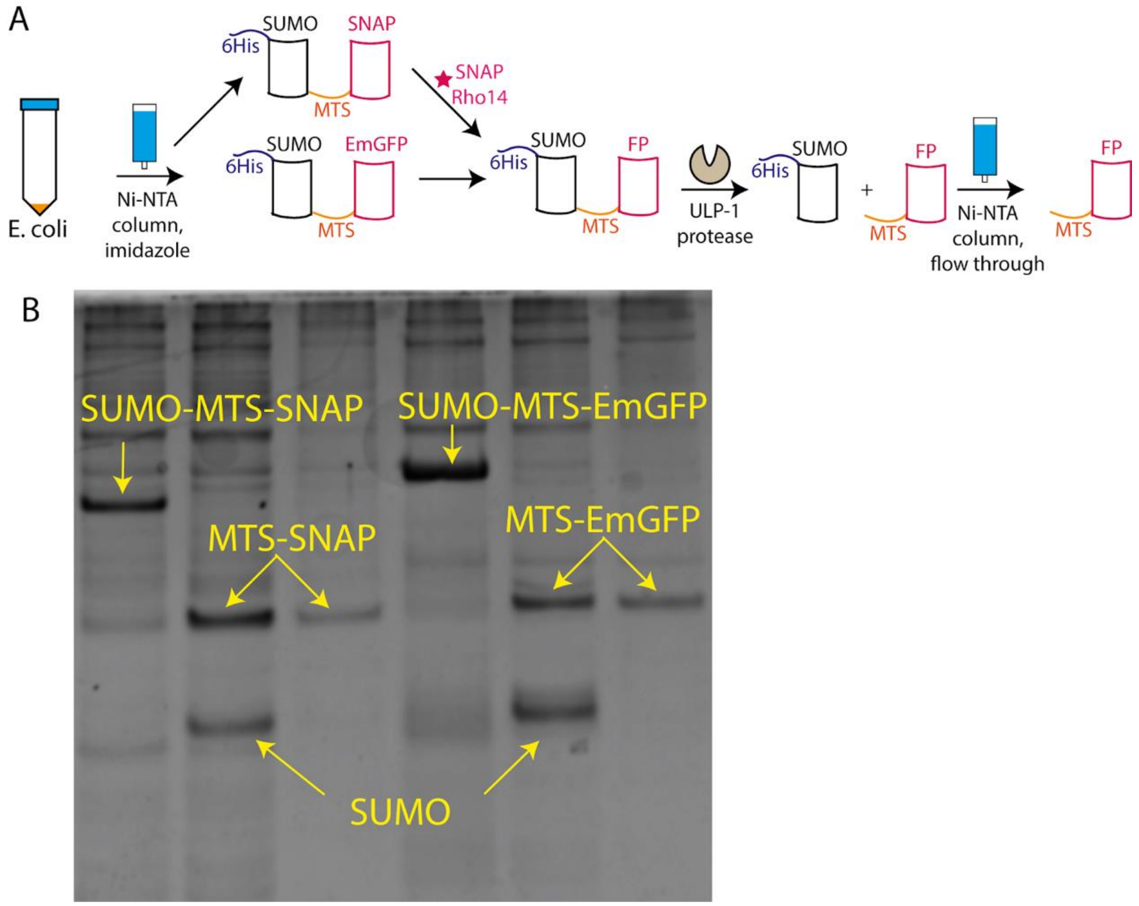

Supplementary Figure 1. (A) Scheme of FP preparation. The initial purification by Ni-NTA is driven by 6His-tag contained in the SUMO protein. After purification (and labeling in the case of the SNAP-tag) SUMO is cleaved by His-tagged protease ULP-1, and the target protein flows through the Ni-NTA column, leaving the SUMO protein and protease on a column. (B) SDS page of the expressed proteins: the SUMO-MTS-SNAP-tag protein after initial purification (1st column), mix of the SUMO and MTS-SNAP-tag proteins after ULP-1 cleavage (2nd column), and the MTS-SNAP-tag protein after final purification (3rd column). The three most right columns depict similar steps for the MTS-EmGFP purification.

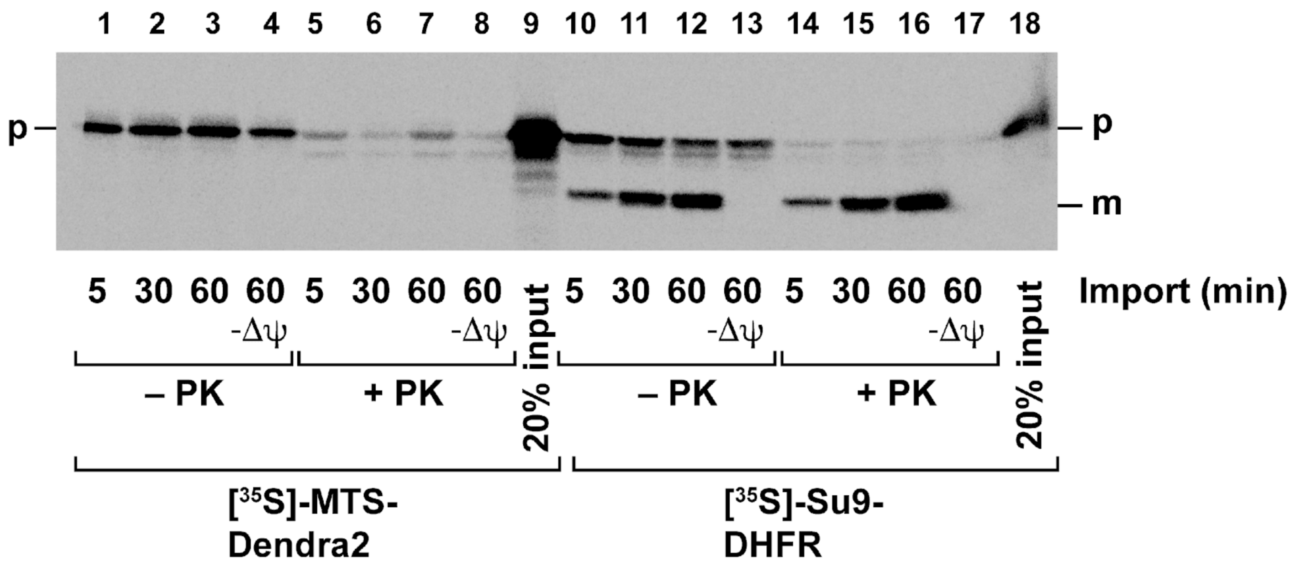

Supplementary Figure 2. Import of the radiolabeled proteins MTS-Dendra2 and Su9-DHFR into isolated yeast mitochondria.35S-labeled proteins were generated by in vitro transcription/translation in reticulocyte lysate and stored at -80° C. The import reactions were performed essentially as described (Becker et al., 2009). The radiolabeled proteins were thawed and added to the intact and energized mitochondria isolated from the yeast cells. The import reactions were performed at 30° C for the indicated times (minutes). In control reactions (-Δψ), the mitochondrial inner membrane potential was depleted. The successful import reactions are characterized by processing of the added precursor (p) form to the mature (m) protein and its resistance against digestion by externally added proteinase K (PK). Both processing and protease resistance do not occur in the absence of a membrane potential. The Su9-DHFR was used as a positive control showing import to the mitochondria.

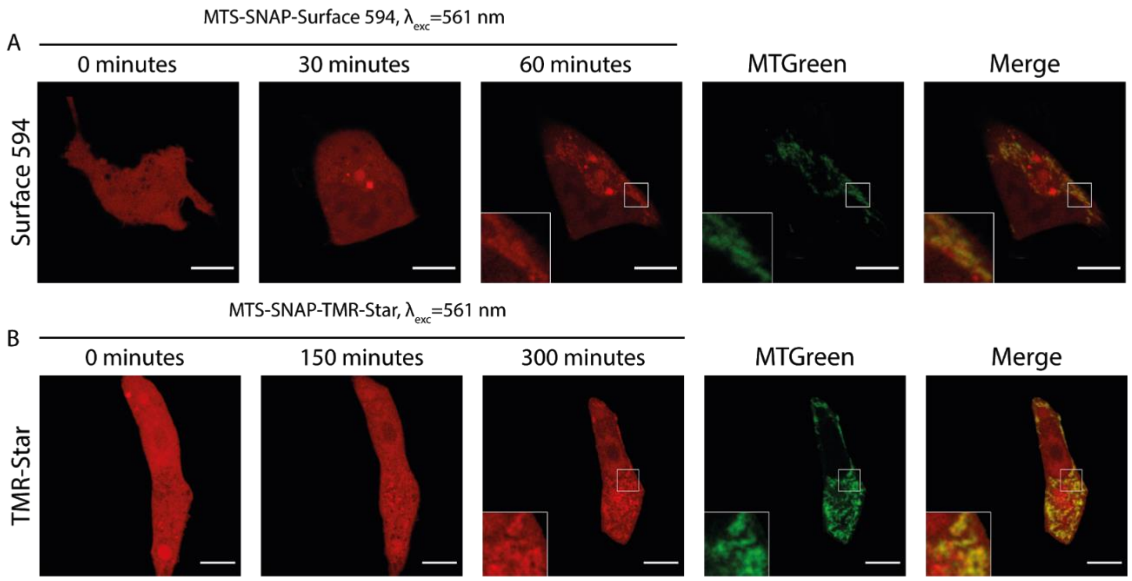

Supplementary Figure 3. Import of MTS-SNAP-tag protein labeled with commercially available dyes functionalized for binding to the SNAP-tag protein. Time-lapse microscopy of the microinjected MTS-SNAP-tag protein (red) import into the mitochondria labeled with the SNAP-Surface594 (A) and SNAP-TMR-Star (B). The mitochondria of HeLa cells are stained with MTGreen (green). SNAP-Surface-594 labeled MTS-SNAP-tag protein is imported with similar characteristic time to the Rho14 labeled protein, while SNAP-TMR-Star labeled MTS-SNAP-tag protein is imported noticeably slower. Scale bar 10 μm.

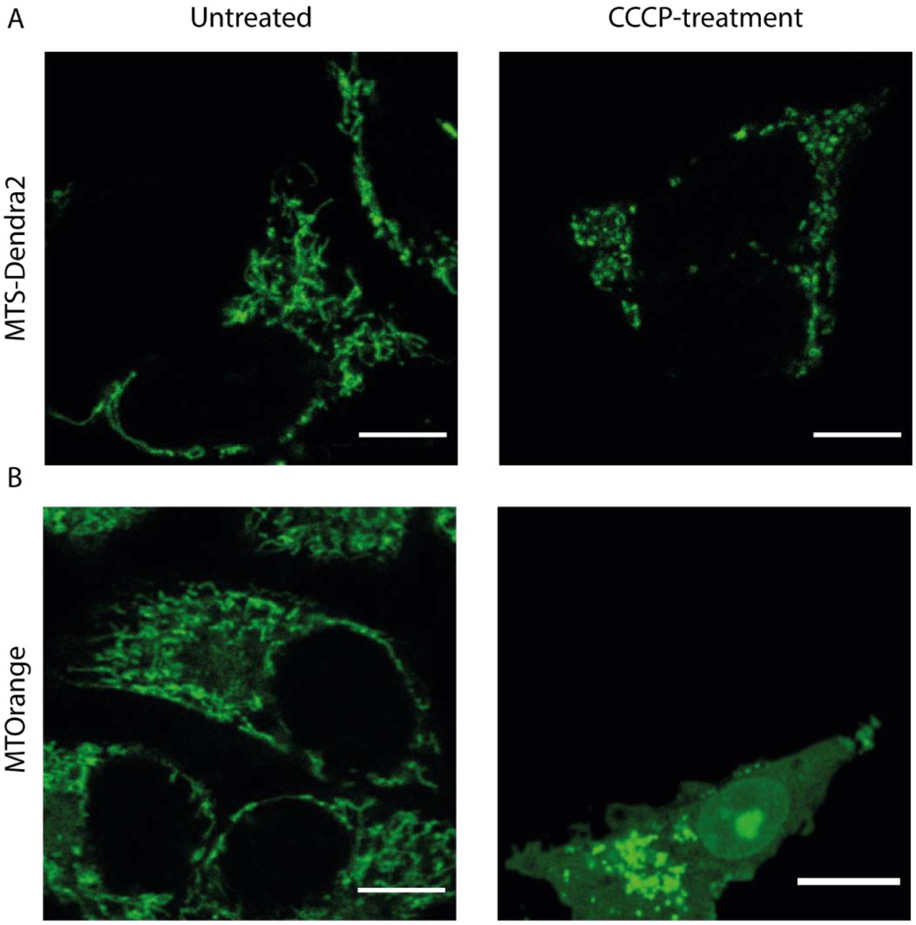

Supplementary Figure 4. Mitochondria staining with MTOrange and MTS-Dendra2 after CCCP treatment. (A) The HEK293 MTS-Dendra2 mitochondria (green). After 50 µM CCCP treatment mitochondrial network fragments, however, Dendra2 remains inside the "mitochondria and mitochondrial network looks unperturbed. (B) The HeLa cells labeled with MTOrange (green). After 50 µM CCCP treatment, MTOrange partially exits the mitochondria, resulting in MTOrange fluorescence from cytosol, nucleus and different cell organelles and a low contrast of the fragmented mitochondria. Scale bar 10 µm.

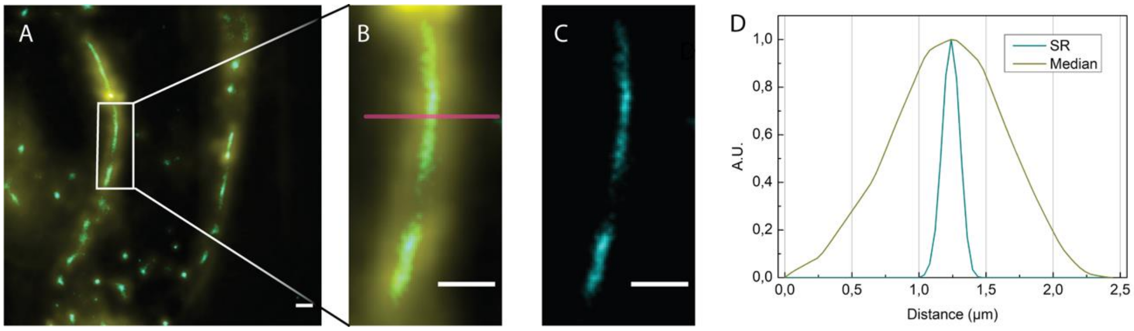

Supplementary Figure 5. Super-resolved image of MTS-SNAP-tag Rho14 localization inside mitochondria. (A) Median wide field image taken over 20 s (1000 frames) (yellow) and reconstructed single molecule image (cyan), scale bar 1 µm. (B) Zoomed in region of the image (A), scale bar 1 µm. (C) Super-resolution image of the mitochondria, scale bar 1 µm. (D) Profile of the normalized intensity over magenta line shown in (B). Average FWHMs of the intensity profiles (spaced by 200 nm) are 170±20 nm/210±30 nm for dSTORM image and 800±60 nm/ 700±70 nm for WF image, for top/bottom mitochondria in (C), respectively.

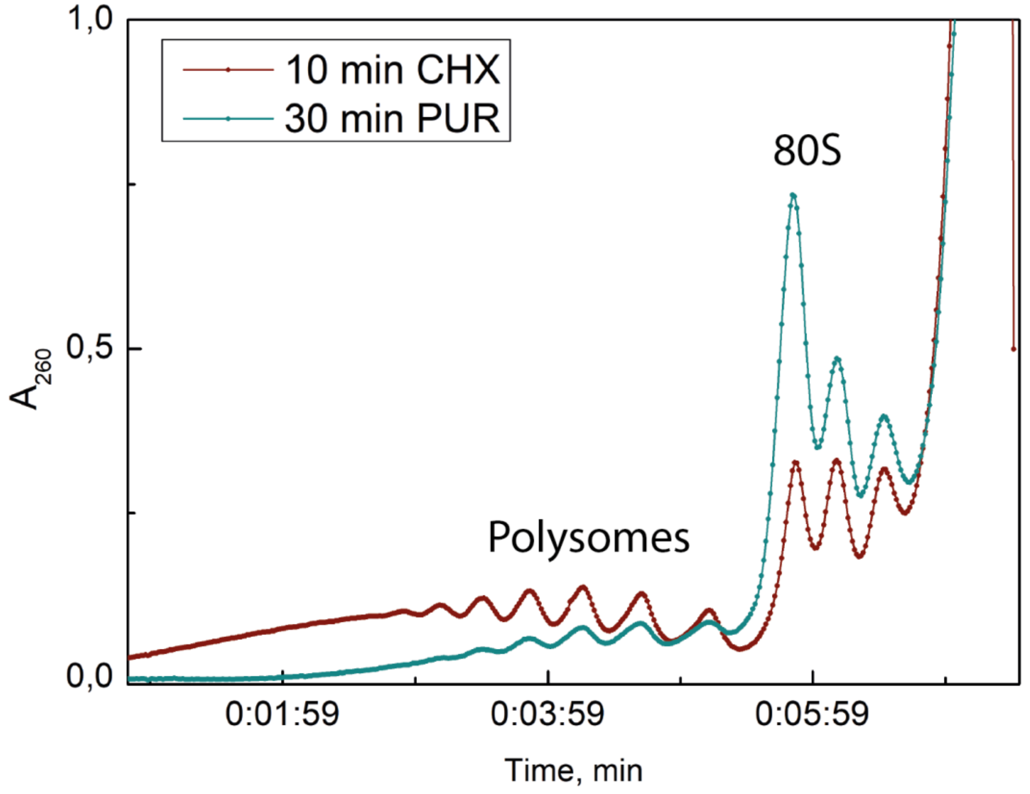

Supplementary Figure 6. Polysome profiles PUR- and CHX-treated HEK293 MTS-Dendra2 cells. Cells were treated for 30 min by 20µg/ml PUR (blue curve) or for 10 min by 100 µg/ml CHX (red curve) (the same as used in corresponding microinjection experiments). PUR treatment causes disassembly of polysomes and accumulation of free ribosomes and their subunits while CHX treatment preserves polysomes.

2 Supplementary video descriptions

Video S1. pMC MTS-EmGFP expression vector injection into the HeLa cells.

The two-channel fluorescence time-series is represented by three windows in the video: the MTOrange labeled mitochondria fluorescence (green, left window), the MTS-EmGFP fluorescence (red, center window), and the merged fluorescence channel (right window). The time between the frames is 5 min.

Video S2. pMC MTS-Dendra2 expression vector injection into the HeLa cells.

The two-channel fluorescence time-series is represented by three windows in the video: the MTOrange labeled mitochondria fluorescence (green, left window), the MTS-Dendra2 fluorescence (red, center window), and the merged fluorescence channel (right window). The time between the frames is 5 min. The protein expression starts only after the completion of cell division about 4 h after the injection.

Video S3. MTS-EmGFP protein injection into the HeLa cells

The two-channel fluorescence time-series is represented by three windows in the video: the MTOrange labeled mitochondria fluorescence (green, left window), the MTS-EmGFP fluorescence (red, center window), and the merged fluorescence channel (right window). The time between the frames is 5 min. The MTS-EmGFP equilibrates between cytosol and nucleus within 1 h, however, the mitochondria continue to be seen as darker regions in the MTS-Em-GFP fluorescence image.

Video S4 MTS-SNAP-tag protein injection into the HeLa cells

The two-channel fluorescence time-series is represented by three windows in the video: the MTOrange labeled mitochondria fluorescence (green, left window), the MTS-SNAP-tag fluorescence (red, center window), and the merged fluorescence channel (right window). The time between the frames is 2 min. The MTS-SNAP-tag protein is imported into the mitochondria over the course of 1 h.

Video S5 MTS-SNAP-tag protein injection into the MTS-Dendra2 HEK293 cell

The two-channel fluorescence time-series is represented by three windows in the video: the MTS-Dendra2 labeled mitochondria fluorescence (green, left window), the MTS-SNAP-tag fluorescence (red, center window), and the merged fluorescence channel (right window). The time between the frames is 2 min. The MTS-SNAP-tag protein import into the mitochondria is seen after 1 h in the MTS-Dendra2 HEK293 cell.

Video S6 SNAP-tag protein injection into the MTS-Dendra2 HEK293 cell.

The two-channel fluorescence time-series is represented by three windows in the video: the MTS-Dendra2 labeled mitochondria fluorescence (green, left window), the SNAP-tag fluorescence (red, center window), and the merged fluorescence channel (right window). The time between the frames is 2 min. The SNAP-tag remains in the cytoplasm over the course of 1 h similar to the injection of the MTS-EmGFP into HeLa cell.

Video S7 MTS-SNAP-tag protein injection into the CCCP-treated MTS-Dendra2 HEK293 cell

The two-channel fluorescence time-series is represented by three windows in the video: the MTS-Dendra2 labeled mitochondria fluorescence (green, left window), the MTS-SNAP-tag fluorescence (red, center window), and the merged fluorescence channel (right window). The time between the frames is 2 min. One noticeable feature in the cytoplasm is the appearance of bright dots of the MTS-SNAP-tag fluorescence. These peculiar dots are not co-localized with the mitochondria and are likely to be protein aggregates in lysosomes and proteasomes.

Video S8 MTS-SNAP-tag protein injection into the CHX-treated MTS-Dendra2 HEK293 cell

The two-channel fluorescence time-series is represented by three windows in the video: the MTS-Dendra2 labeled mitochondria fluorescence (green, left window), the MTS-SNAP-tag fluorescence (red, center window), and the merged fluorescence channel (right window). The time between the frames is 2 min. The MTS-SNAP-tag protein import is clearly seen after 1 h.

Video S9 MTS-SNAP-tag protein injection into the PUR-treated MTS-Dendra2 HEK293 cell

The two-channel fluorescence time-series is represented by three windows in the video: the MTS-Dendra2 labeled mitochondria fluorescence (green, left window), the MTS-SNAP-tag fluorescence (red, center window), and the merged fluorescence channel (right window). The time between the frames is 2 min. The mitochondria become noticeable in the protein channel at approximately 10 min and significantly brighter at 20 min, significantly faster compared to the import in the CHX-treated or untreated cells.
